# Ivory Harvesting Pressure on the Genome of the African Elephant: A Phenotypic Shift to Tusklessness

**DOI:** 10.1007/s12105-016-0704-y

**Published:** 2016-02-26

**Authors:** Erich J. Raubenheimer, Hilde D. Miniggio

**Affiliations:** Pathology and Oral Biology, School of Oral Health Sciences, Sefako Makgatho Health Sciences University, Pretoria, 0204 South Africa

**Keywords:** Ivory, Dentin, Tusklessness, African elephant

## Abstract

The unique chequered pattern of elephant ivory has made it a desired commodity for the production of various works of art. The demand however outstrips the supply and with soaring prices, illegal tusk harvesting is thriving on the African continent. Formal restrictions placed on trade in elephant products have been ineffective in reversing the rapid decline in elephant numbers. We are presently facing the reality of extinction of free roaming elephant on the African continent. This paper describes the histogenesis of the chequered pattern, the genomic impact of ivory harvesting on the phenotype of breeding herds, and the contribution of science to tracing the origin of illegal ivory.

## Introduction

**Fig. 1:**
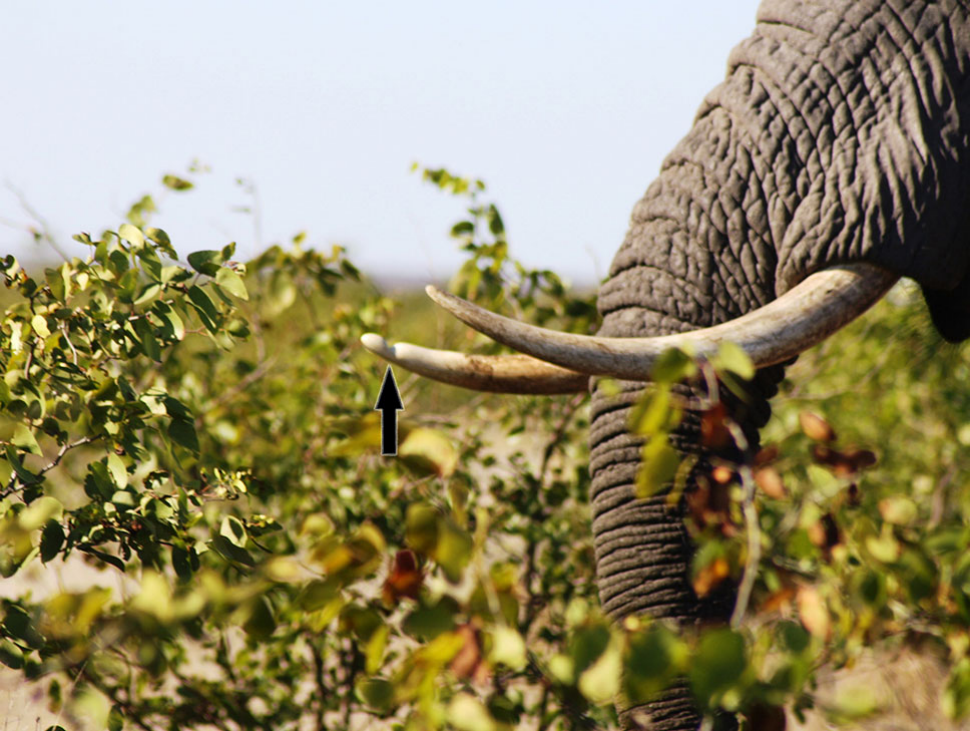
A right tusked elephant cow identified by the notch in the ivory (*arrow*) which is the result of abrasion caused by branches which are clamped with the trunk over the tip of the tusk and ripped off the tree during browsing

With an impressive weight of up to 6 tonnes and shoulder height of 3.6 m, the African elephant (*Loxodonta africana*) is the largest terrestrial animal. Unlike its Asian counterpart, *Elephas maximus*, most African elephant bear ivory tusks, which represent maxillary lateral incisor teeth. The tusk grows continuously throughout life and in older animals a tusk mass in excess of 35 kg is often achieved. Elephant are either right- or left-tusked and the working tusk is revealed by signs of abrasion (Fig. 1). The bulk of a tusk is solid and consists of ivory (or dentin) covered by a thin sheath of dental cementum. A cone shaped pulpal cavity with a large open apex extends a few inches past the skin fold which is around the girth of the tusk [1].

Over the past 16 years, more than half of elephant territory has been lost through human inhabitation and with unabated ivory harvesting and trophy hunting their numbers have dropped significantly in recent years. The estimated 3–5 million African elephant in the early part of the twentieth century has been reduced to barely 300,000 today [2]. This alarming decline is driven by the illegal trade in ivory mainly with countries in the East, where ivory can fetch up to $ 3000 a pound [3]. The demand for ivory drives a trade worth approximately one billion dollars a year [3]. On the African continent where law enforcement is lacking, the income from this low risk, high reward activity enrich corrupt individuals and finance rebel armies involved in civil war. Prior to 1989, an estimated 600–1160 tonnes of ivory were harvested from the continent in 1 year, culminating in the slaughtering of 50,000 elephant annually [4]. This rapid decline elicited a delayed response by the Convention in Trade in Endangered Species (CITES) in 1989 and, despite the introduction of stringent decrees which were modified several times to date, law enforcement has faltered [5] and movement of ivory from the continent still proceeds unabated. The short term survival of elephant in the African continent is in jeopardy. We have become desensitized spectators to the extinction of free roaming elephant on the African continent which is increasingly becoming inevitable.

## Impact of Ivory Harvesting on the Ivory Genome

The key to grasping the adaptation in the phenotypic expression of elephant resulting from the undue pressure on the ivory bearing genome is an understanding of the social structure of the breeding herd. The cow with the largest tusks fends most successfully for her hierarchal position as matriarch. The size of the tusks determines her status and also the status of the other cows in the pecking order and breeding pattern of the herd [1]. In a natural environment free of human interference, congenital absence of a tusk, which follows a gender linked inherited pattern, affects 4.61 % of newborn female cows [6]. A tuskless cow ranks the lowest in the hierarchal order of the herd and due to the dominance of the tusked cows and in particular through the role of the matriarch, they fail to enter the reproductive cycle. It is well established that behavioural experience influences the hypothalamus thereby altering the secretion of hypothalamic releasing factors and pituitary gland function [7]. It is therefore conceivable that emotional dominance could influence the production of pituitary hormones that control the estrous cycle. In this way the ivory bearing genome is maintained and a cohort of elephant is preserved which can effectively fend for the safety of the herd, remove bark from trees, and dig for water and salt, joint ventures which shape the survival of the herd.

The immediate effect of trophy hunting and ivory harvesting, which removes the large tuskers from the herd, is devastating. The sound of rifles discharging, low frequency calls of distress inaudible to the human ear, and the smell of blood scatters the closely knit herd over several square miles. The sounds of calves calling for their caretakers attract carnivores which feast on those who have lost their protection and are unable to fend for their survival, as regrouping of the herd may take several days (personal observation). The expansion of the herd is set back by more than the loss of one dominant tusked cow.

The longer term effect of removing a large tusker and disturbing the social hierarchy and discipline within a herd results in tuskless females unnaturally entering the reproductive cycle [6]. It requires several months to re-establish a new social hierarchal order and during this period the phenotypic shift towards tusklessness is induced. The percentage of tuskless elephant provides a fairly accurate assessment of the pressure brought about by ivory harvesting within a herd. In the Kruger National Park where hunting is prohibited, approximately 3 % of elephant are tuskless, compared to 10 % in Mana Pools in Zimbabwe and more than 70 % in the Addo Elephant Park in the Eastern Cape [8]. The selection for tusklessness in the Addo elephant population is the result of intense ivory harvesting during the nineteenth century when the populations first made contact with humans with modern rifles and commercial interests. Three thousand years of exposure of the Asian elephant, *E. maximus* to man hunter, have virtually eradicated the ivory bearing gene and the majority of calves today are born without tusks [1, 9]. This genetic shift, which is undoubtedly taking place in Africa, is the only glimmer of hope for the long term survival of *Loxodonta africana* as tuskless animals are less attractive to poachers and hunters alike.

## Why Elephant Ivory?

**Fig. 2:**
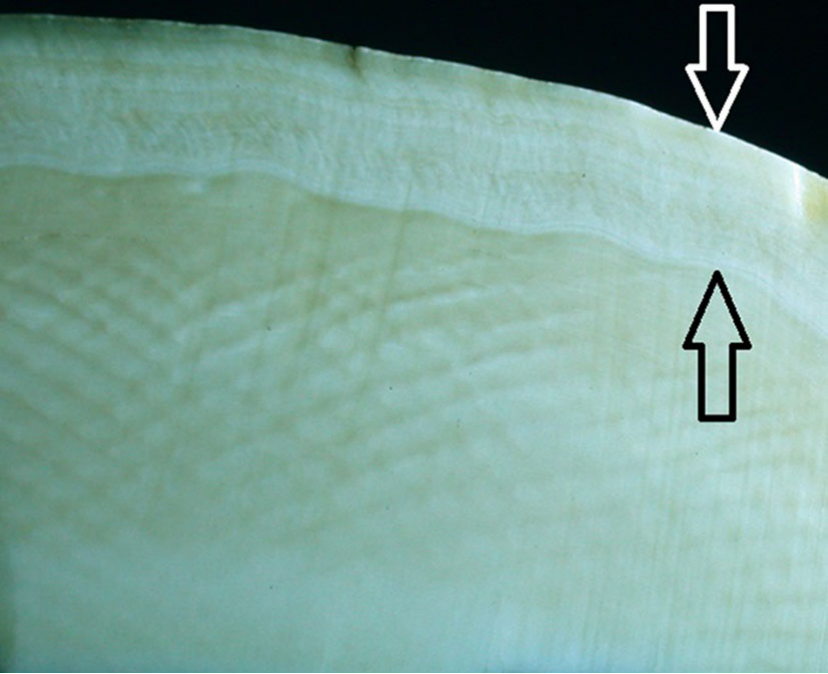
Polished surface of a cross section through a tusk showing the chequered pattern of ivory. *The arrows* indicate the outer sheath of cementum

**Fig. 3:**
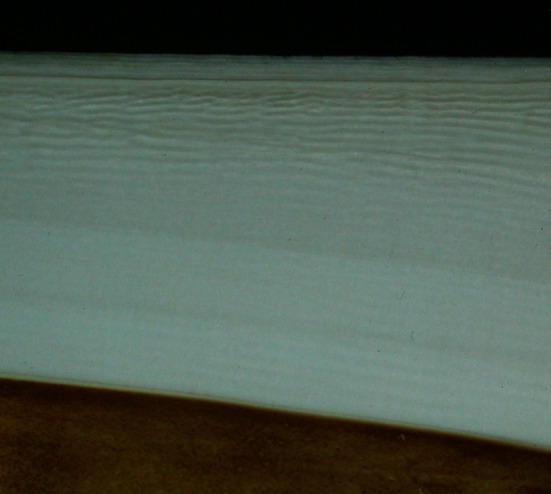
Polished surface of a sagittal section through a tusk demonstrating the parallel and alternating *light* and *dark bands* in the ivory. The peripheral cementum sheath is towards the *top* of the image

**Fig. 4:**
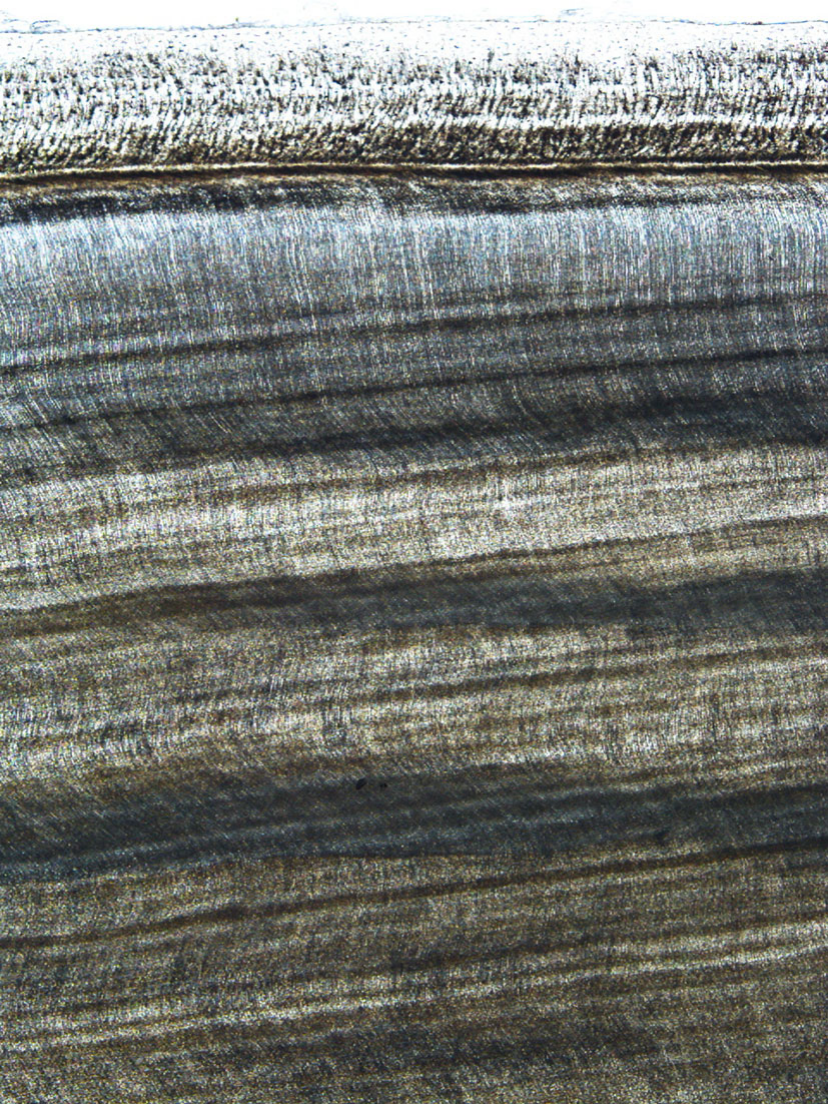
Microscopic appearance of the regular sweeping sinusoidal curve followed by the odontoblastic tubules. The *dark bands* represent the posteriorly directed sector of the curve where the tubules are closer packed. The outer sheath of cementum is towards the *top* of the image (unstained section ×200)

**Fig. 5:**
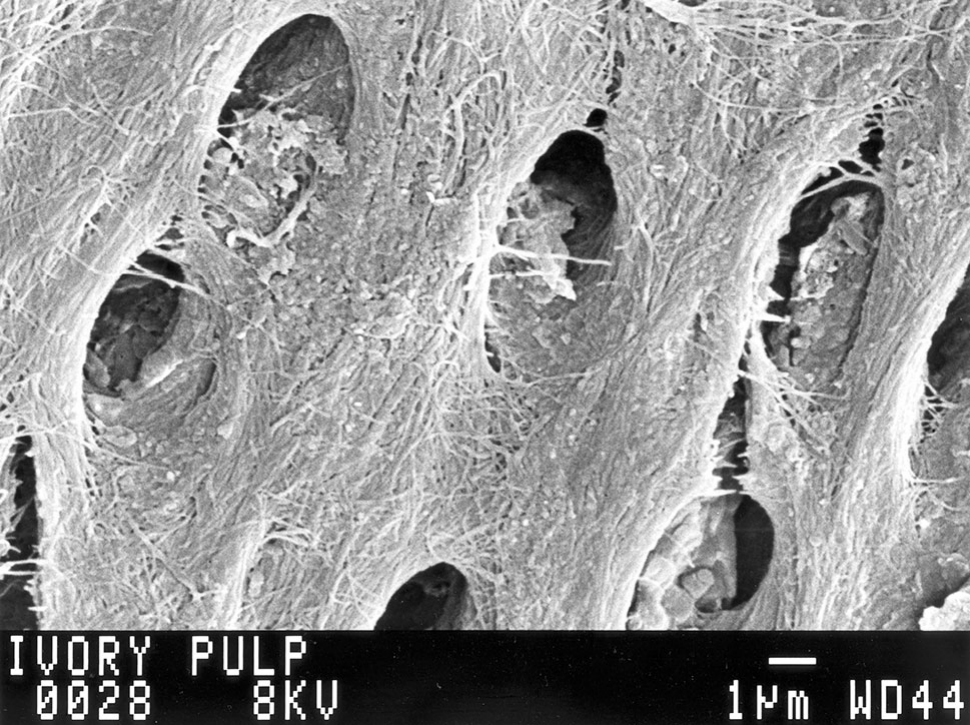
Scanning electron micrograph of the pulpal openings of odontoblastic tubules. *Note* the anterior-to-posterior flattening of the tubules which is the result of odontoblastic compression due to crowding during their centripetal course

There are several large tuskers such as hippopotamus, warthog, and narwhales in the animal kingdom, yet there is no demand for their ivory. The reason for this is that elephant ivory is unique in that it demonstrates a regular chequered pattern on polished surfaces prepared perpendicular to the axis of the tusk (Fig. 2). Since the early days of man, this pattern has made elephant ivory a precious commodity and medium for artistic expression in many cultures. On polished surfaces prepared along the axis of the tusk, the pattern corresponds with alternating light and dark bands which is the optical manifestation of the sinusoidal curve followed by the odontoblastic tubules through the full thickness of ivory (Fig. 3). As the odontoblasts move along their centripetal course during dentinogenesis, the circumference of the pulp becomes smaller and the odontoblastic layer experience crowding with increasing compression of cells. The cell mass gradually moves towards the posterior opening of the conical pulpal cavity, the tubules become closer spaced and a dark band develops. With a further increase in the pressure brought about by the centripetal movement, apoptosis and fusion of odontoblasts occur which alleviates the compression caused by crowding, and the spaces between tubules subsequently increase, resulting in a light band and forward slanting of the tubules [10]. Due to the thickness of elephant ivory, this process results in a sinusoidal passage that repeats itself several times (Fig. 4). The pulpal openings of the tubules are oval due to crowding and compression of the odontoblasts (Fig. 5). The primary curve of odontoblastic tubules in human teeth does not complete a full cycle due to the lesser thickness of the human dentine. The micro-tubular arrangement, with between 40 and 60 thousand tubules per square millimetre, provides an explanation for the preference of elephant ivory for the construction of piano keys as their capillary action maintains dry fingertips. The dark lines with their higher density of tubules are “weak” regions in ivory and fractures follow these lines, exposing the underlying pattern on the fracture surface. The reasons for the absence of this unique pattern in the ivories of other tusk bearing animals are speculative but possibly related to a more rapid reduction of odontoblastic numbers during their centripetal movement suspending the crowding phenomenon, thereby resulting in odontoblastic tubules that follow a more linear course with subsequently a more homogenous macroscopic appearance.

## Composition of Ivory

Scientific studies on the composition of ivory contributed significantly to the provision of baseline data for the identification of the site of origin of illegal ivory. The carbon isotope ratios (^13^C:^12^C) distinguish between elephant roaming woodlands and those in dense forests [11], the ratio between the nitrogen isotopes (^15^N:^14^N) are related to water stress and rainfall and strontium isotope ratios (^87^Sr:^86^Sr) reflect the geology of a particular region [12]. The determination of the stable isotope series in elephant ivory reveals extensive seasonal and annual variations in dietary niches within a herd, but little variation between them [13]. Studies in our laboratory demonstrated a total of 20 elements in elephant ivory which varied significantly in concentrations in ivory obtained from different elephant sanctuaries in Southern Africa [14]. Unlike bone, the composition of ivory remains stable throughout life and can be used not only to identify the geographic site of origin of a tusk, but also monitor environmental pollution. Determination of the distribution of isotopes and trace elements in ivory with mass spectrometry in different areas of a single tusk provides valuable information on the migratory patterns of elephant across different geological regions. Analyses of the organic fraction demonstrated 17 amino acids in ivory [14]. Tusks from arid regions where elephant feed mainly on dry vegetation show low proline and hydroxyproline content and under hydroxylation of lysine which corresponds with the increased brittleness of their tusks. These changes affect the strength of the collagen scaffold of the mineralized apatite [15] and occurs amongst others in scurvy as vitamin C is an important cofactor in the hydroxylation of amino acids during the biosynthesis of the tropocollagen molecule [16]. A vitamin C deficiency in the desert elephant is endorsed by the well described craving for fresh fruit of these elephant.

## Conclusion

The unique chequered pattern of African elephant ivory has made it a sought after product that has brought about the inexorable ivory trade which is impacting on their survival. Conceivably, the artificial genetic drift to tusklessness could serve as the biological change that may provide hope for the survival of this splendid animal. This genetic shift, the only glimmer of hope left for the survival of the African elephant, would, nonetheless be suboptimal, rendering the herds unable to dig for water, feed adequately, or fend for themselves. Africa will indeed be impoverished by the disappearance of the tusked phenotype.
